# p16 expression correlates with basal-like triple-negative breast carcinoma

**DOI:** 10.3332/ecancer.2013.317

**Published:** 2013-05-14

**Authors:** Amany A Abou-Bakr, Hany I Eldweny

**Affiliations:** 1 Department of Pathology, National Cancer Institute, Cairo University, Egypt; 2 Department of Surgery, National Cancer Institute, Cairo University, Egypt

**Keywords:** Basal-like, breast cancer, p16

## Abstract

**Background:**

Basal-like breast carcinoma (BLBC) has attracted considerable attention over the past few years. It has been suggested that tumours expressing basal markers have a more aggressive clinical behaviour. However, a molecular basis for this disease remains unclear, and it lacks currently used therapeutic targets. Therefore developing a novel treatment strategy is crucial for improving the prognosis. The aim of this study was to characterise the immunohistochemical (IHC) expression of p16 in patients with BLBC compared with non-BLBC.

**Materials and methods:**

Eighty-five cases of grade-3 invasive ductal carcinomas not otherwise specified (IDC-NOS) were analyzed. Immunohistochemical stains for oestrogen receptor (ER), progesterone receptor (PR), human epidermal growth factor receptor type 2 (HER2), cytokeratin (CK) 5/6, epidermal growth factor receptor (EGFR) and p16 were performed. BLBC was defined as ER-, PR-, Her2- and CK5/6+, and/or EGFR+.

**Results:**

Twenty cases were categorised as BLBC versus 65 as non-basal. High mitotic count and presence of necrosis were associated with basal-like phenotype. Distant metastasis developed in 40% of cases of BLBC with frequent spread to brain and lung. p16 had significantly higher expression in the basal subgroup (80% versus 50.8%, *P *= 0.04). Patients with BLBCs were found to have a lower disease-free survival (DFS) rate (60% versus 70.8%, *P* = 0.03).

**Conclusion:**

BLBC typically demonstrates a unique profile. p16 is frequently expressed in breast cancers with basal-like phenotype; this suggests that p16 may play a role in the poor prognosis of this tumour, and it may be used in the development of a targeted therapy that will result in improved patient prognostication and outcome.

## Introduction

Breast carcinomas are considered to be a heterogeneous group of tumours showing different behaviour, prognosis, and response to treatment [1]. Furthermore, tumours classified under the same histological type and grade can present distinct molecular aspects and biological course. The molecular heterogeneity of breast tumours cannot be morphologically assessed, and it represents an important challenge for the research and treatment of breast cancer [2]. Recently, gene expression profiling analyses using DNA microarrays and later on immunohistochemical (IHC) studies have enabled the recognition of distinct subtypes of tumours associated with different clinical outcomes [3–5]: luminal A (positive for oestrogen receptor (ER) and progesterone receptor (PR) and negative for human epidermal growth factor receptor type 2 [HER2]); luminal B (ER+, PR+, HER2+); HER2 overexpressing (ER-, PR-, HER2+); basal-like (differentiation towards basal cell types); unclassifiable or normal breast-like (tumours that are negative for all the markers above).

There is no consensus on how to define basal-like breast carcinoma (BLBC) based on immunohistochemistry. The majority of BLBCs lack the expression of ER, PR, and HER2 protein overexpression [4, 6, 7]. BLBC has also been characterised by the expression of cytokeratin (CK) 5/6 and 17, epidermal growth factor receptor (EGFR), c-kit, and vascular endothelial growth factor [7, 8, 9]. Nielsen *et al *[7] have developed an IHC panel for identifying BLBCs on the basis of a comparison between the transcriptomic and IHC profiles. According to this definition, BLBCs are negative for ER and HER2 and positive for CK5/6 and/or EGFR. Conversely, others have proposed that a proportion of BLBCs may be positive for ER and HER2 [10, 11].

The prevalence of BLBC ranges from 7.5% to 36.7% of breast cancer cases in different patient cohorts [3, 5, 7, 12–23]. These tumours were predominantly grade-3, displaying a high mitotic count, tumour necrosis, pushing margin of invasion, stromal lymphocytic response, high rates of nuclear pleomorphism, and a lack of tubule formation [24–26].

Basal-like breast cancer remains a great challenge because of its clinically aggressive nature and poorly characterised molecular pathogenesis. Unlike ER-positive luminal tumours and HER2-positive tumours, the basal-like subtype typically lacks expression of the molecular targets that confer responsiveness to highly effective targeted therapies such as tamoxifen, aromatase inhibitors, or trastuzumab. Indeed, identification of the relevant molecular targets in BLBC remains a formidable challenge.

The cyclin-dependent kinase inhibitor-2A gene (p16INK4a), located within the CDKN2A locus, is a strong and specific inhibitor of the progression through the G1 phase of the cell cycle by preventing phosphorylation of Rb protein [27] and is considered a major tumour suppressor gene. The inactivation of p16 seems a crucial event in the development of several human tumours [28]. Hui *et al *[29] demonstrated an inverse relationship between p16INK4a and ER mRNA levels in cell lines and primary breast cancers, suggesting that p16INK4a inactivation by hypermethylation and overexpression is a marker of poor prognosis. Similarly, Milde-Langosch *et al *[30] have described high p16INK4a reactivity (both nuclear and cytoplasmic) as indicative of a more undifferentiated phenotype in mammary carcinomas.

The aim of this study was to characterise the IHC expression of p16 in patients with BLBC compared with non-BLBC in a group of grade-3 invasive ductal carcinoma not otherwise specified (IDC-NOS) to identify a more homogenous subset to which targeted therapy can be applied.

## Material and methods

### Tissue samples

Eighty-five randomly selected cases that underwent surgical treatment and reported as grade-3 IDC-NOS (a subgroup with a traditionally poor prognosis) were analysed in this work. The study protocol was in accordance with the ethical guidelines of the 2004 Declaration of Helsinki. Patients with primary tumours for which paraffin blocks and follow-up data were available were retrieved from the Department of Surgical Pathology, National Cancer Institute, Cairo University, Egypt between January 2000 and January 2005. Special histologic subtypes were not included to avoid any confounding effect, and none of the patients had received adjuvant therapy before surgery. Tumour morphologic parameters including mitotic activity, necrosis, and Nottingham grade [31] were evaluated.

### Immunohistochemistry

Four micron tissue sections were deparaffinised in xylene and rehydrated through a series of decreasing ethanol concentration. The slides were pretreated with hydrogen peroxide (3%) for 10 minutes to remove the endogenous peroxidase, followed by antigen retrieval in microwave for 15 minutes in 10 mM citrate buffer (pH 6.0). The primary antibodies were applied, followed by washing and incubation with the biotinylated secondary antibody for 30 minutes at room temperature. The slides were counterstained with haematoxylin and dehydrated in alcohol and xylene before mounting. Appropriate positive and negative controls were included with each IHC run. The characteristics of the primary antibodies used in this study are listed in Table 1.

### Evaluation of IHC Staining

Tumours were considered to be positive for ER and PR when nuclear positivity was observed in >1% of neoplastic cells [32]. HER2 scoring was performed according to the UK accepted diagnosis criteria [33]. Patients with scores of 0 and 1+ were considered negative, whereas 3+ positive. Equivocal cases (2+) were referred for fluorescence *in situ *hybridisation (FISH) using the Vysis PathVysion HER2 DNA probe kit (Abbott Molecular, Inc, Abbott Park, IL) [34].

For CK5/6, and EGFR > 10% cytoplasmic and/or membranous staining was considered positive [35]. Cases were recorded positive for p16 based on the presence of nuclear and/or cytoplasmic reactivity in >10% of tumour cells [36] with strong intensity. p16 evaluation was done blinded to BLBC category.

In this study, we defined BLBC using the criteria of Carey *et al *and others by the negativity to ER, PR, and HER2 plus the expression CK5/6 and/or EGFR [7, 13, 37].

### Statistical Analysis

Statistical analysis was performed using the computer software StatView (Abacus Concepts, Inc.). Chi-square and Fisher exact tests were used to study the statistical association between categorical variables. Unpaired *t *test was used for analysis of continuous variables. Disease-free survival (DFS) times were estimated in months from the date of diagnosis to the date of relapse, death, or last follow-up. Survival curves were plotted using the Kaplan–Meier method, and differences in survival curves were assessed by the log-rank test. Statistical significance was defined as a *P* < 0.05.

## Results

**Figure 1:**
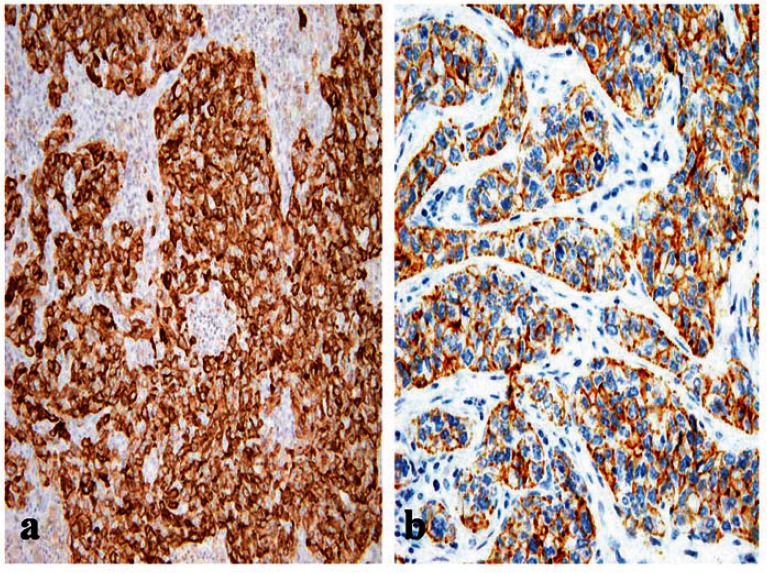
A BLBC that is positive for (a) EGFR (x200) and for (b) CK5/6 (x400).

Of the 85 cases, 20 had a basal-like breast cancer profile performed (Figure 1). They occurred at a slightly, but significantly, younger age than other grade-3 tumours. Although we found a higher proportion of premenopausal women (65%) in the basal-like phenotype, this difference was not statistically significant (*P* = 0.46).

There were no statistically significant differences regarding primary tumour size, presence of vascular invasion and tumour stage (*P *> 0.05). BLBC was less likely to have axillary nodal metastasis at diagnosis (*P* = 0.05).

**Figure 2:**
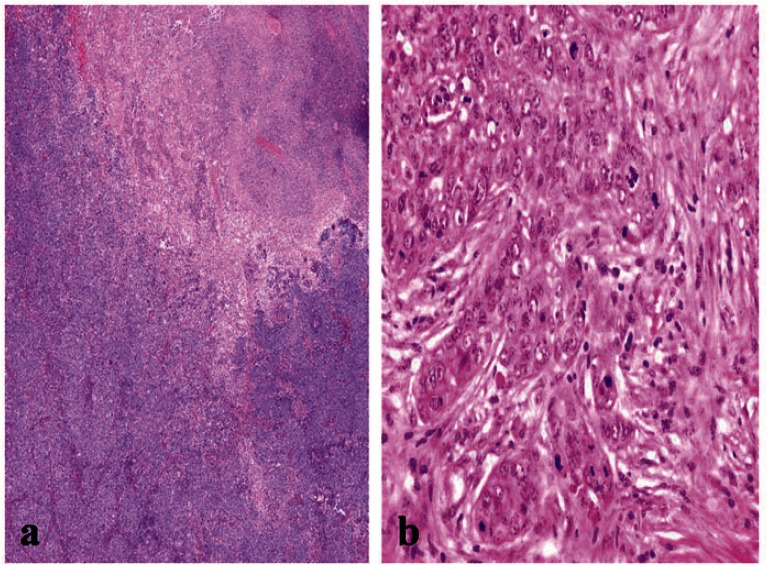
A BLBC showing (a) central necrosis (x200), and (b) sheets of markedly pleomorphic cells with conspicuous mitosis (x400).

The presence of necrosis and mitotic count (Figure 2) showed differences between the two groups. Necrosis was more prevalent in the basal-like tumour group compared with the non-basal group (65% versus 35.4%, *P *= 0.04). A difference was also apparent between the mean mitotic counts, the non-basal mean (15 mitosis/10 high-power fields [HPF], range 8–50) was significantly lower than that of the basal-like group (30 mitosis/10 HPF, range 10–70).

**Figure 3:**
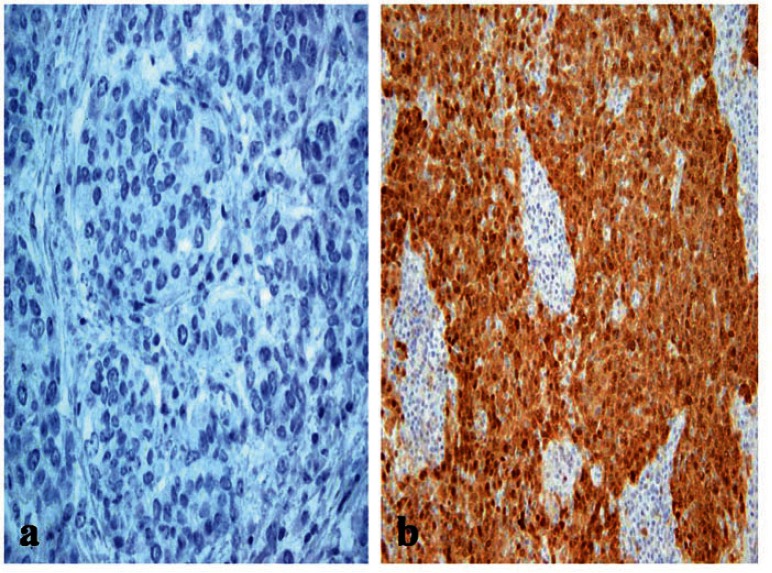
A p16 stain revealing (a) negative reaction in non-BLBC (x400) and (b) diffuse cytoplasmic/nuclear positivity in BLBC (x200).

The frequency of p16 positive cases was higher in the BLBC (Figure 3). p16 was expressed in 16/20 (80%) in basal cancers and in 33/65 (50.8%) of non-BLBC with a significant difference (*P *= 0.04). In the basal category, positive cases demonstrated labelling in >75% of neoplastic cells with a mean of 81%, while the mean was 32% (range 10%–90%) in non-basal tumours.

## Follow-up, outcome and sites of metastases

Average time of follow-up was 37.7 months (ranging from six to 60 months). In the whole cohort, 41 patients developed loco-regional or distant relapse, among these 23 patients died of breast cancer.

In BLBC, local recurrence was detected in three cases (15%), whereas systemic relapse was reported in 8/20 patients (40%), brain and lungs being the main sites (30% and 25%, respectively). Other sites of metastases were liver (10%), bone (10%) and non-regional lymph nodes (15%). There were clear differences between the basal and non-basal tumours in relation to distant metastatic sites (Table 2). Basal tumours were more likely to develop brain and lung metastases (*P *= 0.03 and 0.05, respectively) but significantly less likely to develop bone (*P* = 0.03) or liver (*P *= 0.04). There was no difference in the rate of involvement of non-regional lymph nodes (*P* = 0.86).

**Figure 4:**
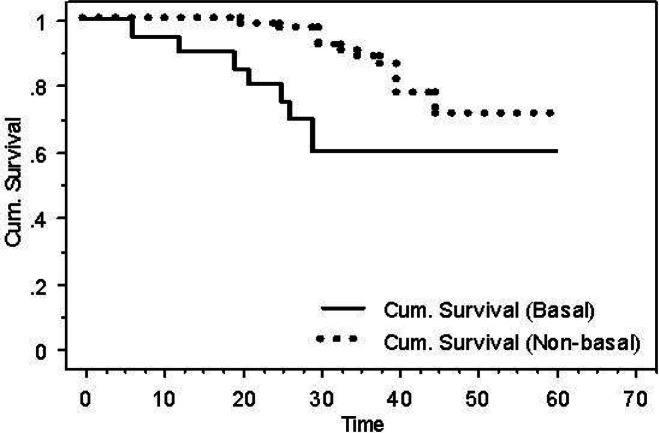
Kaplan-Meier DFS for basal versus non-basal tumours (*P* = 0.03).

After performing a log-rank test, survival analyses showed that patients with BLBC had a worse DFS when compared with patients with non-basal tumours (60% versus 70.8%, *P* = 0.03) (Figure 4).

## Discussion

Basal-like tumours are gaining an increasing amount of attention in part owing to recognition as a distinct entity, but most importantly owing to the overall poor prognosis that the diagnosis indicates. In this study, BLBC was associated with shorter DFS. Most gene profiling studies have repeatedly reported a shorter metastasis free and overall survival among basal breast cancer patients [3, 12, 14, 16, 17, 38–42]. According to three different multigene expression signatures, most of the tumours predicted as poor prognosis were basal [43]. Data are variable with IHC [42], possibly because the terminology and definitions surrounding the concept of basal tumours are controversial, and a plethora of different markers have been employed to identify cases in clinical studies.

Also our results revealed that approximately 40% of basal-like carcinomas developed distant metastasis, more often to brain and lung than to the liver or bone [44–49]. These findings suggest that basal-like tumours might possess a distinct mechanism of metastatic spread. In fact, our observations together with the absence of association with lymph node involvement, or loco-regional relapse do not appear to justify a more radical approach to local or axillary surgery. The potentially aggressive behavior of these tumours may be better approached by the development of new systemic therapeutic strategies and targeting molecular alterations. Recent clinical trials are currently focusing on identifying these possible targets. Therefore, the main objective of this study was to examine p16 expression in basal phenotype to help in defining molecular features of this breast cancer subset.

p16 protein overexpression has been shown to be associated with breast carcinomas having poor prognostic factors [29, 30]. Importantly, virtually all of these studies occurred before the entity of basal-like cancer was established by gene expression profiling in 2001. Since the recognition of this entity, the p16 status of BLBC has not been systematically assessed. One study [50] indicated that BLBC associated with BRCA1 gene inactivation expresses lower levels of p16 than carcinomas associated with inactivation of BRCA2, which were more frequently ER positive. In addition, a review of BLBC- and BRCA1-associated tumours also states that these tumours are characterised by lower levels of p16 than the typical breast carcinoma [51]. In contrast, Gauthier *et al *[52] in the setting of a study of ductal carcinoma *in situ *noticed that BLBC show high levels of p16 transcripts and low levels of Rb transcripts. Analysing a subset of primary tumours representing each of the five molecular subtypes of breast cancer, they showed that p16 IHC labelling correlated well with mRNA levels in BLBC, although they did not statistically compare their p16 results in BLBC with those of the other different molecular subtypes. They hypothesised that loss of functional p16/Rb signalling may play a defining role in the biology of this tumour subtype. Our results correlate with their hypothesis where BLBC demonstrated significantly higher frequency of p16 positivity compared with non-BLBC.

Similarly Rakha *et al*’s results [21] support this and showed a difference in the expression of cell cycle regulators between basal and non-basal triple-negative tumours with p16 protein being expressed at high levels by BLBC. In accordance Bohn *et al *[53] found a strong positive reaction with p16 antibody in 16 out of 18 (89%) basal cases. They propose p16 as a biomarker for identification of truly basal-like cancers and raise the possibility that triple-negative breast cancer with basal cytokeratin, and p16 co-expression may adequately identify these tumours and serve as a potential diagnostic/prognostic biomarker.

Subhawong *et al *[54] examined the Rb/p16 pathway in BLBC because of their distinct morphologic similarities to human papilloma virus (HPV)-related poorly differentiated squamous cell carcinomas (SCCs). On the basis of similar morphology, they hypothesised that similar genes may be inactivated in these neoplasms, albeit by different mechanisms (genomic or epigenetic alterations in breast cancer, HPV in SCCs). Their data demonstrated Rb-/p16+ immunophenotype in 15 of 21 BLBCs and 9 of 12 unclassifiable triple-negative carcinomas, but only 1 of 14 HER2-positive cases and none of the 17 luminal A or 7 luminal B cases (*P *< 0.01). So they suggested that BLBC represents a non-HPV-related carcinoma in which basal-like morphology predicts inactivation of Rb protein and diffuse p16 expression.

Another intriguing link between p16INK4a and basal-like cells comes from studies on human mammary epithelial cells, which have been shown to resemble the basal-like subtype by gene expression analysis [55, 56]. It was suggested that RB1 recruits Polycomb repression complexes to the p16INK4a locus, which silence p16INK4a transcription [57].

In summary, we suggest that p16 may define a more homogenous subgroup of BLBC, which allow further stratification of these tumours enabling more efficacious therapeutic strategy for individual patients and lessen the burden of over treatment.

## Conclusion

BLBC are a distinct clinical and pathological group representing about 25% of grade-3 invasive ductal carcinomas. They show a specific pattern of distant metastasis with an increased propensity for metastases to brain and lung, sites known to be associated with a poorer prognosis. p16 is a frequent occurrence in these cancers and may play a role in the poor outcome; therefore it might be used as a potential therapeutic target to alter the clinical course of the disease and improve the survival for individual patients. Further studies on a larger cohort are needed to confirm these findings.

## Figures and Tables

**Table 1:** Characteristics of the primary antibodies.

Marker	Clone	Species	Manufacturer	Dilution
ER	Sp1	Rabbit mAb	Dako	Ready to use
PR	PGR636	Mouse mAb	Dako	Ready to use
HER2	CB11	Mouse mAb	Dako	Ready to use
CK5/6	D5/16B4	Mouse mAb	Dako	Ready to use
EGFR	E30	Mouse mAb	Dako	1:100
p16	16p07	Mouse mAb	NeoMarkers	1:25

**Table 2:** Clinicopathologic characteristics of the studied cases.

Variable	BLBC No (%)	Non-BLBC No (%)	*P* value
**Age**			
Mean ± SD, years	44±8	48.3±9	0.03
**Menopausal status**			
Pre	13 (65%)	34 (52.3%)	0.46
Post	7 (35%)	31 (47.7%)	
**Tumour size**			
Mean ± SD, cm	3.6±1.9	4.5±2.2	0.10
**Lymph node**			
Positive	8 (40%)	44 (67.7%)	0.05
Negative	12 (60%)	21 (32.3%)	
**Lymphovascular invasion**			
Yes	9 (45%)	39 (60%)	0.35
No	11(55%)	26 (40%)	
**Stage**			
I	1(5%)	2 (3.1%)	0.78
II	6 (30%)	10 (15.4%)	0.26
III	10 (50%)	40 (61.5%)	0.51
VI	3 (15%)	13 (20%)	0.86
**Necrosis**			
Yes	13 (65%)	23 (35.4%)	0.04
No	7 (35%)	42 (64.6%)	
**Mitotic count**			
Mean ± SD, mitosis/10HPF	30±10	15±8	<0.0001
**P16**			
Positive	16 (80%)	33 (50.8%)	0.04
Negative	4 (20%)	32 (49.2%)	
Loco-regional recurrence	3 (15%)	13 (20%)	0.86
Systemic metastasis	8 (40%)	17 (26.2%)	0.36
**Systemic metastasis site**			
Brain	6 (30%)	5 (7.7%)	0.03
Lung	5 (25%)	4 (6.2%)	0.05
Liver	2 (10%)	24 (36.9%)	0.04
Bone	2 (10%)	25 (38.5%)	0.03
Non regional LN	3 (15%)	13 (20%)	0.86
